# A Novel Role for the DNA Repair Enzyme 8-Oxoguanine DNA Glycosylase in Adipogenesis

**DOI:** 10.3390/ijms22031152

**Published:** 2021-01-25

**Authors:** Sai Santosh Babu Komakula, Bhavya Blaze, Hong Ye, Agnieszka Dobrzyn, Harini Sampath

**Affiliations:** 1 Rutgers Center for Lipid Research, New Jersey Institute for Food, Nutrition, and Health, Rutgers University, New Brunswick, NJ 08901, USA; saisanthosh36@gmail.com (S.S.B.K.); bhavya.blaze@rutgers.edu (B.B.); hy296@rutgers.edu (H.Y.); 2 Laboratory of Cell Signaling and Metabolic Disorders, Nencki Institute of Experimental Biology, 02-093 Warsaw, Poland; a.dobrzyn@nencki.gov.pl; 3 Department of Nutritional Sciences, Rutgers University, New Brunswick, NJ 08901, USA; 4 Center for Microbiome, Nutrition, and Health, New Jersey Institute for Food, Nutrition, and Health, Rutgers University, New Brunswick, NJ 08901, USA

**Keywords:** adipocyte differentiation, DNA repair, base excision repair, lipid accretion, obesity

## Abstract

Cells sustain constant oxidative stress from both exogenous and endogenous sources. When unmitigated by antioxidant defenses, reactive oxygen species damage cellular macromolecules, including DNA. Oxidative lesions in both nuclear and mitochondrial DNA are repaired via the base excision repair (BER) pathway, initiated by DNA glycosylases. We have previously demonstrated that the BER glycosylase 8-oxoguanine DNA glycosylase (OGG1) plays a novel role in body weight maintenance and regulation of adiposity. Specifically, mice lacking OGG1 (*Ogg1^−/−^*) are prone to increased fat accumulation with age and consumption of hypercaloric diets. Conversely, transgenic animals with mitochondrially-targeted overexpression of OGG1 (*Ogg1^Tg^*) are resistant to age- and diet-induced obesity. Given these phenotypes of altered adiposity in the context of OGG1 genotype, we sought to determine if OGG1 plays a cell-intrinsic role in adipocyte maturation and lipid accumulation. Here, we report that preadipocytes from *Ogg1^−/−^* mice differentiate more efficiently and accumulate more lipids than those from wild-type animals. Conversely, OGG1 overexpression significantly blunts adipogenic differentiation and lipid accretion in both pre-adipocytes from *Ogg1^Tg^* mice, as well as in 3T3-L1 cells with adenovirus-mediated OGG1 overexpression. Mechanistically, changes in adipogenesis are accompanied by significant alterations in cellular PARylation, corresponding with OGG1 genotype. Specifically, deletion of OGG1 reduces protein PARylation, concomitant with increased adipogenic differentiation, while OGG1 overexpression significantly increases PARylation and blunts adipogenesis. Collectively, these data indicate a novel role for OGG1 in modulating adipocyte differentiation and lipid accretion. These findings have important implications to our knowledge of the fundamental process of adipocyte differentiation, as well as to our understanding of lipid-related diseases such as obesity.

## 1. Introduction

Nuclear and mitochondrial DNA constantly face oxidative DNA damage through reaction with endogenous and exogenous oxidants. 8-Oxo-7,8-dihydroguanine (8-oxoG) is the most commonly formed oxidative DNA lesion in the cell. Due to its propensity to mispair with adenines during replication, unrepaired guanine lesions can lead to mutagenesis and cellular transformation [1,2,3,4]. Furthermore, the presence of oxidized guanines in GC-rich promoter regions have been implicated in altering gene transcription, thereby impacting cellular function [5,6,7,8]. 8-oxoG is primarily excised by the base excision repair (BER) glycosylase OGG1, which localizes to both the nucleus and mitochondria [9,10,11,12,13]. Modulation of OGG1 activity has been implicated in numerous disease pathways, including cancers [14,15,16,17,18,19,20,21] and neurological diseases such as Parkinson’s [22,23,24] and Alzheimer’s disease [1,25,26,27,28,29]. Further, we have reported a novel role for OGG1 in regulating metabolic health. Specifically, mice with a global deletion of the *Ogg1* gene (*Ogg1^−/−^*) are prone to both age-induced and diet-induced obesity and adiposity [30,31,32]. *Ogg1^−/−^* mice display increased adipose mass along with significant increases in hepatic and serum lipids, concomitant with marked glucose intolerance, skeletal muscle atrophy, and adipocyte inflammation [30,31,32]. Consistent with these findings in a mouse model, human cohort studies indicate that polymorphisms in the *OGG1* gene are directly associated with increased incidence of obesity, type II diabetes, and mortality due to cardiovascular disease [33,34,35].

In contrast to obesity and glucose intolerance resulting from OGG1 deficiency, we recently reported that constitutive overexpression of mitochondrially-targeted human OGG1 significantly protected mice from high-fat diet induced obesity and adiposity [36]. This metabolic protection was associated with key alterations in adipose tissue of OGG1-overexpressing transgenic (*Ogg1^Tg^*) mice. Specifically, adipocyte size and markers of adipose inflammation were significantly reduced, and levels of the metabolically protective adipokine adiponectin were increased in *Ogg1^Tg^* mice [36]. In addition, mitochondrial content and respiration were both significantly increased in adipose tissue of *Ogg1^Tg^* animals [36]. Given these intriguing changes in adipose tissue of *Ogg1^Tg^* mice, we were interested in determining if the DNA repair protein OGG1 plays a cell-intrinsic role in the adipocyte, particularly in the process of differentiation and subsequent lipid accumulation. To address this question, we used adipocytes in culture to delineate the function of OGG1 in the adipocyte for the first time. Using both the well-established 3T3-L1 pre-adipocyte fibroblast cell line, as well as stromal vascular cells isolated from *Ogg1^Tg^* and *Ogg1^−/−^* mice, we report here our discovery of a novel role for OGG1 in differentiation and lipid accretion in the adipocyte.

## 2. Results

### 2.1. OGG1 Expression and Activity Increases during Adipogenesis

**Figure 1:**
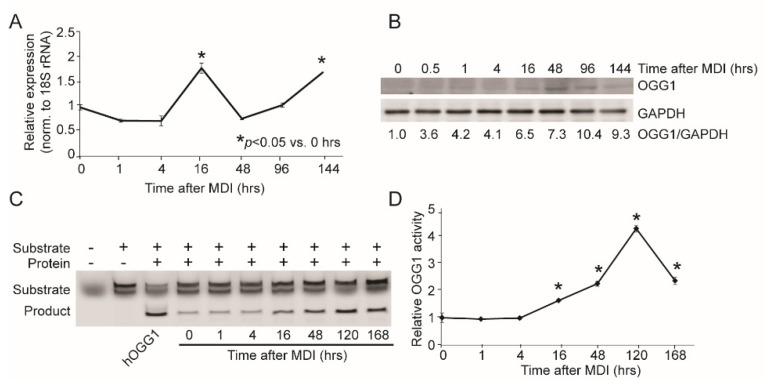
OGG1 expression and activity are regulated during adipogenesis. 3T3-L1 cells were differentiated using MDI. Cells were harvested, and RNA and proteins were isolated at indicated time points. (**A**) Gene expression of *Ogg1* was measured by qRT-PCR using 18S rRNA as a control. (**B**) OGG1 protein expression was analyzed by Western blotting. (**C**,**D**) Proteins isolated at the indicated time points were used for the OGG1 activity assay. Data are expressed as average ±  SEM and represent at least 3 independent replicates per time point. * *p*  <  0.05 vs. 0 h.

Virtually nothing is known regarding the expression and regulation of OGG1 during the adipogenic process. We therefore measured the gene and protein expression of OGG1, as well as OGG1 activity, at early and late time points during adipogenic differentiation of 3T3-L1 cells. *Ogg1* gene expression was significantly increased very early in differentiation, 16 h following induction of differentiation (Figure 1A). Following a brief decline in gene expression at 48 h, *Ogg1* expression continued to increase robustly throughout the differentiation period (Figure 1A). Immunoblotting also confirmed a continuous increase in OGG1 protein expression during adipogenic differentiation (Figure 1B). Corresponding with protein expression, OGG1 activity steadily increased throughout the differentiation period, beginning at 16 h (Figure 1C,D). Interestingly, this pattern of regulation was not observed for other BER glycosylases such as Neil-like DNA glycosylase 1 (*Neil1)*, *Neil2*, or endonuclease III-like protein 1 (*Nth1*), but a similar early induction of gene expression was observed for *Neil3* (Supplementary Figure S1).

### 2.2. Ogg1^−/−^ Preadipocytes Differentiate More Efficiently, While Ogg1^Tg^ Cells Have Reduced Lipid Accretion

**Figure 2:**
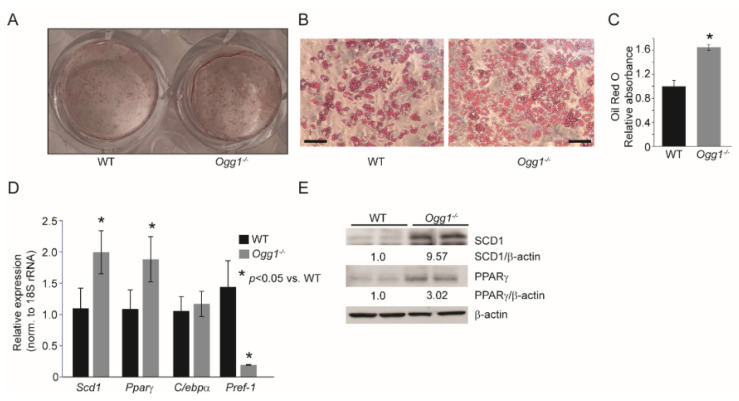
Primary adipocytes from *Ogg1^−/−^* mice accumulate more lipid than WT cells. Preadipocytes isolated from WT and *Ogg1^−/−^* mice were differentiated using MDI. (**A**–**C**) On the 6th day of differentiation, cells were stained with Oil Red O, and RNA and protein were isolated for (**D**) gene expression analyses and (**E**) immunoblotting. The scale bar in Figure 2B represents 130 µm. Data are expressed as average ±  SEM and represent at least 3 independent replicates per time point. * *p*  <  0.05 vs. 0 h.

**Figure 3:**
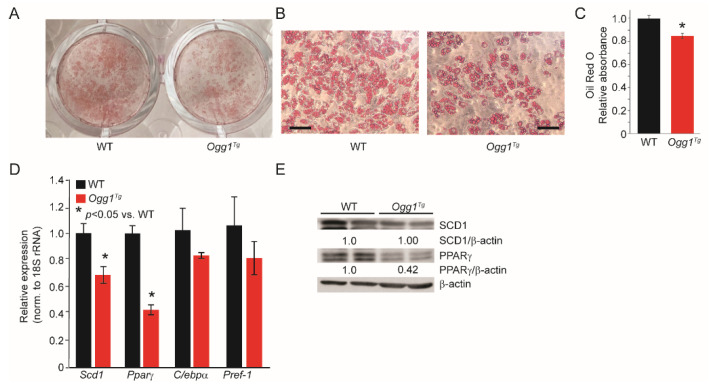
Primary adipocytes from *Ogg1^Tg^* mice accumulate less lipid than WT cells. Preadipocytes isolated from WT and *Ogg1^Tg^* mice were differentiated using MDI. On the 8th day of differentiation, (**A**–**C**) cells were stained with Oil Red O, and RNA and protein were isolated for (**D**) gene expression analyses and (**E**) immunoblotting. The scale bar in Figure 3B represents 130 µm. Data are expressed as average ±  SEM and represent at least 3 independent replicates per time point. * *p*  <  0.05 vs. 0 h.

To understand the role of OGG1 in adipogenesis, preadipocytes were isolated from the stromal vascular fraction (SVF) of wild-type (WT), *Ogg1^−/−^*, and *Ogg1^Tg^* cells, and differentiated in culture using 3-isobutyl-1-methylxanthine (IBMX), dexamethasone, and insulin (MDI). Cells from *Ogg1^−/−^* mice accumulated significantly more lipids compared to cells derived from WT counterparts (Figure 2A–C). This was accompanied by a significant increase in expression of terminal markers of adipocyte differentiation in *Ogg1^−/−^* cells, including peroxisome proliferator activated receptor-gamma (PPARγ) and stearoyl-CoA desaturase-1 (SCD1) (Figure 2D,E). Conversely, expression of preadipocyte factor-1 (*Pref-1*), an inhibitor of adipogenic differentiation, was significantly reduced in *Ogg1^−/−^* cells (Figure 2D), corresponding with the observed increase in lipid accumulation (Figure 2A,B). In contrast, preadipocytes isolated from *Ogg1^Tg^* mice accumulated significantly less lipids compared to WT cells (Figure 3A–C). Consistent with this reduced lipid accretion, differentiated adipocytes from *Ogg1^Tg^* mice had reduced gene expression of the terminal differentiation markers *Pparγ*, *Scd1*, and *C/ebpα* (Figure 3D). Protein expression of PPARγ was reduced in *Ogg1^Tg^* cells, relative to WT controls (Figure 3E), consistent with reduced adipogenic differentiation.

### 2.3. OGG1 Overexpression Inhibits Adipogenic Differentiation and Lipid Accumulation in 3T3-L1 Cells

**Figure 4:**
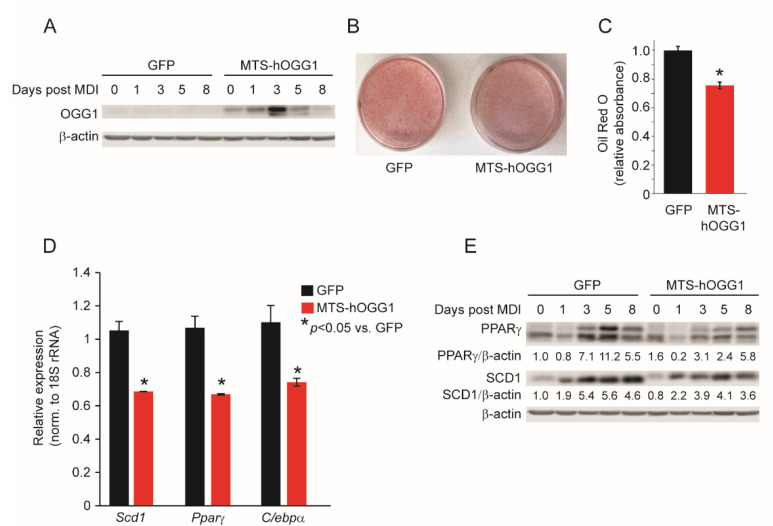
Overexpression of MTS-hOGG1a impairs adipogenic differentiation in 3T3-L1 cells. 3T3-L1-CARΔ cells were transduced with adenovirus particles to overexpress either GFP or MTS-hOGG1-1a and differentiated using MDI. (**A**) OGG1 expression was assessed by immunoblotting at the indicated time points. On the 8th day of differentiation, (**B**,**C**) cells were stained with oil Red O, and RNA was isolated for (**D**) gene expression analyses. (**E**) Proteins were isolated at the indicated time points for immunoblotting. Data are expressed as average ±  SEM and represent at least 3 independent replicates per time point. * *p*  <  0.05 vs. 0 h.

To further evaluate a cell-intrinsic role for OGG1 in adipogenic differentiation, we transduced 3T3-L1 CARΔ cells [37] with an adenoviral plasmid expressing mitochondrially-targeted GFP-tagged human OGG1a (MTS-hOGG1 cells) [37]. Control 3T3 CARΔ cells were transduced with GFP-expressing plasmids (GFP cells) (Figure S2). 3T3-L1 CARΔ cells are stably modified through the introduction of the gene-encoding coxsackie and adenovirus receptor (CAR), which allows for approximately 100-fold more efficient transduction than parental 3T3-L1 cells [37]. Adipogenic differentiation was induced two days after adenoviral transduction. Immunoblotting for OGG1 confirmed persistent overexpression of OGG1 during the adipogenic differentiation process (Figure 4A). After eight days of differentiation, Oil red O staining revealed that overexpression of MTS-hOGG1a significantly reduced lipid accumulation in 3T3 cells, relative to GFP overexpression (Figure 4B,C). These results were consistent with our observations of reduced lipid accretion in adipocytes derived from *Ogg1^Tg^* animals (Figure 3A). Consistent with reduced lipid accumulation, gene expression of *Pparγ*, *Scd1*, and *C/ebpα* was significantly reduced in MTS-hOGG1 adipocytes (Figure 4D). MTS-hOGG1 adipocytes also showed lower protein expression of PPARγ and SCD1 during the course of differentiation, indicating delayed and reduced expression of terminal adipocyte differentiation markers in these cells (Figure 4E).

### 2.4. OGG1 Alters Cellular PARylation in Adipocytes

**Figure 5:**
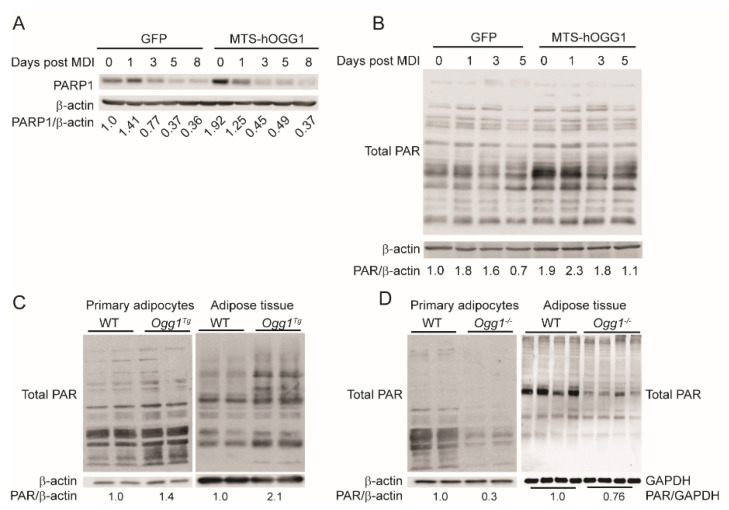
OGG1 genotype alters PARylation of cellular proteins in adipocytes and adipose tissue. Proteins were isolated from 3T3-L1-CARΔ cells transduced with GFP or MTS-hOGG1-1a for detection of (**A**) PARP1 and (**B**) total cellular PARylation by immunoblotting. Cellular PARylation was assessed in protein extracts from differentiated primary adipocytes and adipose tissue from (**C**) WT vs. *Ogg1^Tg^* animals and (**D**) from WT vs. *Ogg1^−/−^* mice. Data are representative of at least 3 independent replicates for cell studies and 4–6 age- and sex-matched animals per genotype for tissue extracts. * *p*  <  0.05 vs. 0 h.

OGG1 is a known activator of the protein poly(ADP-ribose) polymerase-1 (PARP1) [38]. Activation of PARP1 results in post-translational modification of proteins via the addition of poly ADP ribose moieties [39]. Recent studies have demonstrated that poly(ADP-ribosyl)ation (PARylation) of cellular proteins inhibits adipogenesis, and a whole-body knockout model of PARP1 was shown to develop obesity and increased adiposity [40,41,42], similar to the *Ogg1^−/−^* mouse [31]. Since the role of OGG1 on PARylation in adipocytes has never been assessed, we asked if the alterations in adipogenesis are associated with differential cellular PARylation. Prior to the start of differentiation, hOGG1 overexpressing cells had significantly higher expression of PARP1, relative to GFP control cells (Figure 5A). We found that PARP1 protein expression rapidly declined in both cell types during the process of differentiation, and by 24 h after initiation of differentiation, there were no significant differences in expression of PARP1 between control and hOGG1 overexpressing cells (Figure 5A). Total cellular PAR levels also declined during the course of adipogenesis, corresponding with PARP1 expression, and as has been previously reported [42]. However, cellular PARylation was higher in MTS-hOGG1 cells at all time points, relative to GFP controls (Figure 5B). These results indicate increased PARP1 activity in OGG1 overexpressing adipocytes and correspond with reduced lipid accretion in these cells. Similar to 3T3-L1 cells, total protein PARylation was also increased in differentiated primary adipocytes from *Ogg1^Tg^* mice (Figure 5C), corresponding with their reduced lipid accretion (Figure 3). Increased cellular PARylation was also observed in adipose tissue protein extracts from *Ogg1^Tg^* mice (Figure 5C), indicating that these alterations persist in vivo and not just in adipocytes differentiated in culture. These increases in PARylation in *Ogg1^Tg^* adipocytes are also consistent with their lean phenotype and smaller adipocyte size, as we have previously reported [36].

Conversely, cellular PAR levels were significantly reduced in primary adipocytes and adipose tissue extracts from *Ogg1^−/−^* mice (Figure 5D). These results are consistent with the observed increases in lipid accretion and adipocyte differentiation in *Ogg1^−/−^* cells (Figure 2). The reduction in cellular PARylation in adipose tissue from *Ogg1^−/−^* mice is also consistent with their propensity to adiposity and obesity, as we have previously reported [31]. Interestingly, similar reductions in cellular PAR levels were also observed in liver and brown adipose tissue extracts from *Ogg1^−/−^* mice (Supplemental Figure S3), indicating a role for OGG1 in activating PARP activity in vivo, consistent with reported observations in vitro. The metabolic and cellular implications of these changes are under investigation.

## 3. Discussion

OGG1 is a bifunctional DNA glycosylase belonging to the EndoIII superfamily of DNA glycosylases. Its primary substrates are 8-oxoguanine (8-oxoG) and the formamidopyrimidine derivative of guanine (FapyG) [30,43]. Both mouse OGG1 and human OGG1a are localized to the nucleus and mitochondria [30,43,44]. However, at least in murine tissues, nuclear OGG1 activity is consistently and significantly higher than mitochondrial activity across tissues (Sampath, unpublished). Overexpression of the human *OGG1a* gene downstream of the mitochondrial targeting sequence from the *MnSOD* gene results in constitutive and significant overexpression of OGG1 (Figure 4A) and has been previously reported to significantly increase mitochondrial OGG1 activity without impacting nuclear activity in diverse cell types [36,45,46]. However, the construct still retains its native nuclear localization sequence, and relative increases in mitochondrial vs. nuclear OGG1 activity were not determined in the current study in adipocytes. Therefore, the effects observed in adipocytes overexpressing this construct cannot at present be attributed solely to the mitochondrial vs. nuclear effects of OGG1. Further studies using constructs that lack the nuclear localization sequence will be required to clarify potential differences.

We have previously reported that the DNA repair enzyme OGG1 plays an unexpected and novel role in the development of obesity and adiposity [31,32,36]. *Ogg1^−/−^* mice are prone to diet-induced obesity and inflammation. Conversely, mice constitutively expressing human OGG1a are significantly resistant to high-fat diet induced obesity. These *Ogg1^Tg^* mice also display significant alterations in adipose tissue metabolism, including increased levels of the adipokine adiponectin, increased mitochondrial content and function, longer and more electron-dense mitochondria in adipose tissue, and reduced adipocyte size [36]. Given these findings in adipose tissue from *Ogg1^Tg^* mice, we asked if OGG1 may play a cell-intrinsic role in the adipocyte. Prior to this investigation, nothing was known regarding a role for this protein in the adipocyte. Our results clearly indicate that OGG1 depletion results in accelerated adipocyte differentiation and increased lipid filling (Figure 2). Conversely, OGG1 overexpression blunts adipocyte differentiation and lipid accretion (Figure 3 and Figure 4). These changes in adipocyte differentiation are evident both in primary adipocytes isolated from inguinal fat depots of *Ogg1^−/−^* and *Ogg1^Tg^* mice, as well as in 3T3-L1 cells overexpressing hOGG1a, thereby increasing the rigor of our findings. This is the first report indicating a role for this DNA repair protein in regulating adipocyte differentiation. Further, we show that the alterations in adipocyte differentiation are accompanied by changes in cellular protein PARylation (Figure 5). OGG1 deficiency is associated with reduced PARylation, while OGG1 overexpression increases cellular PAR-levels. These changes are not accompanied by changes in PARP1 protein expression, and are consistent with a reported role for OGG1 in activating PARP1 in mouse embryonic fibroblasts [38]. To our knowledge, this is also the first report of differences in PARylation in tissue extracts, particularly in adipose tissue, in mice with altered OGG1 genotype.

In addition to OGG1, the BER glycosylase NEIL1 has also been reported to bind to and activate PARP1 [47]. While we did not observe any changes in *Neil1* expression during the adipogenic process, a similar obesity susceptibility phenotype has been reported in mice lacking NEIL1 [48,49]. Thus, it will be of interest to determine if NEIL1 inhibition is also associated with increased adipocyte differentiation and lipid accretion, analogous to OGG1 inhibition. It is also of interest to note that of the BER glycosylases examined, *Neil3*, showed a similar pattern of expression as *Ogg1* during the adipogenic process (Figure S1). Both genes were upregulated at early time points, unlike other glycosylases measured. NEIL3 is a bifunctional DNA glycosylase with broad substrate specificity and activity against both oxidized purines and pyrimidines, but not 8-oxoG. NEIL3 has been shown to excise further oxidized products of 8-oxoG, including spiroiminodihydantoin (Sp) and guanodinohydantoin [50,51]. Virtually nothing is known regarding a potential role for the NEIL3 glycosylase in modulating adipogenesis or metabolic function. However, the similar regulation of both *Ogg1* and *Neil3* in differentiating adipocytes suggests a potentially novel role for NEIL3 in adipogenic differentiation and perhaps in adipose tissue function.

Our studies indicate an inverse correlation between cellular PARylation levels and adipogenic differentiation capacity, as well as obesity resistance. There are conflicting reports regarding the role of PARP1 activity in regulating body weight. Two different models of whole body PARP1 deletion reported opposing results regarding either protection from or exacerbation of obesity as a consequence of PARP1 deletion [40,52], while a more recent report indicated that PARP1 ablation only in preadipocytes resulted in the development of obesity [41]. The reasons for these discrepancies are not yet clear, but may be related to the genetic background of the animal model and tissue-specific differences in the consequences of PARP1 deletion, with regard to whole animal metabolism [53]. Our studies demonstrate that the increased propensity to obesity in *Ogg1^−/−^* mice is associated with reductions in PARP1 activity, while obesity resistance in *Ogg1^Tg^* animals is associated with increased cellular PARylation. We also note that these apparent changes in PARP1 activity are evident not only in white adipose tissue, but also in other metabolically active organs, such as brown adipose tissue and liver (Figure S3). The role of altered cellular PARylation in mechanistically mediating the metabolic phenotypes of *Ogg1^−/−^* and *Ogg1^Tg^* mice are yet to be elucidated.

Separately from the issue of body weight regulation, PARP1 has also been demonstrated to influence adipogenesis [41]. A previous report indicated that inhibition of PARP1 either via siRNA-based depletion or by chemical inhibition resulted in increased adipocyte differentiation and lipid accretion [42]. This was mediated by alterations in PARylation of the early adipogenic factor, CEBPβ, which regulates subsequent expression of factors such as CEBPα and PPARγ [42], both of which are modulated differentially by OGG1 status (Figure 2, Figure 3 and Figure 4). A more recent report indicated that activation of PARP1 by small nucleolar RNA inhibited adipogenesis [41]. Together with these results, our findings of increased cellular PARylation in *Ogg1^Tg^* adipocytes that differentiate less effectively and decreased PARylation in *Ogg1^−/−^* adipocytes that accumulate more lipids are suggestive of an inhibitory role for PARP1 activity in adipogenesis.

In summary, using both primary adipocytes from *Ogg1^−/−^* and *Ogg1^Tg^* mice, as well as 3T3-L1 cells transfected in culture, our studies establish for the first time a novel role for the DNA repair protein OGG1 in modulating adipocyte differentiation and lipid accumulation. These results are consistent with previous reports of obesity susceptibility in OGG1-deficient mice, decreased lipid accumulation in mice overexpressing OGG1, and reported correlations between OGG1 polymorphisms and obesity in human cohorts. They thus add to our growing understanding of the role of this protein in regulating metabolic homeostasis.

## 4. Material and Methods

### 4.1. Animals

The generation of *Ogg1^Tg^* and *Ogg1^−/−^* mice has been previously described [10,31,32,36,45]. Both strains were backcrossed for over ten generations to the C57BL/6J background. The breeding and care of animals are in accordance with the protocols approved by the Animal Care and Use Committee of Rutgers University, New Brunswick, NJ, USA (Protocol ID: 201900077; Latest approval date: 10/28/2020). Five to six male WT, *Ogg1^Tg^*, and *Ogg1^−/−^* mice aged between 5–6 weeks old were used to isolate cells from SVF of inguinal white adipose tissue (iWAT). Four to six 12-week old chow-fed males were used for tissue immunoblotting experiments.

### 4.2. Isolation of Preadipocytes from the Stromal Vascular Fraction (SVF)

Mice SVF was isolated as previously described [54]. Briefly, mice (5–6 weeks old) were euthanized, and iWAT was collected in Hank’s balanced salt solution (HBSS), supplemented with 1% penicillin and streptomycin. Collagenase-I (0.2%) solution was prepared in HBSS supplemented with 1% penicillin and streptomycin. Adipose tissue was minced, followed by digestion in collagenase-I solution for 1 h at 37 °C. Digested tissues were filtered through 100 µm strainers and centrifuged at 800× *g* for 10 min. The cell pellet was treated with red blood cell lysis buffer (Sigma-Aldrich. St. Louis, MO, USA) for 1 min and centrifuged at 800× *g* for 4 min. The resulting cell pellet was resuspended and cultured in DMEM/F12 medium supplemented with 10% FBS and 1% penicillin and streptomycin.

### 4.3. Adipocyte Differentiation

Pre-adipocytes were grown until 2 days post confluency before initiating adipogenic differentiation. Cells were incubated with DMEM-F12 medium supplemented with 10% FBS, 1% penicillin and streptomycin, 10 µg/mL insulin, 50 mM IBMX, and 1 µM dexamethasone (all from Sigma-Aldrich) for 2 days. The medium was changed to DMEM/F12 supplemented with 10% FBS, 1% penicillin and streptomycin, 1 µg/mL insulin for the next 4 days. 3T3-L1 and 3T3-L1 CARΔ cells were grown until 2 days post confluency before addition of differentiation medium containing high glucose DMEM supplemented with 10% FBS, 1% penicillin and streptomycin, 10 µg/mL insulin, 50 mM IBMX, and 10 μM dexamethasone. For adenoviral transductions, cells were transduced 2 days prior to induction of differentiation with the methylisobutylxanthine, dexamethasone, insulin (MDI) medium for 3 days. The medium was then changed to high glucose medium supplemented with 10% FBS, 1% penicillin and streptomycin for the next 5 days.

### 4.4. Oil Red O Staining

Oil red O stock solution (0.35%) was prepared and filtered using a 0.22 µm filter. A working solution (0.21%) was prepared by dissolving Oil red O stock in distilled water, followed by filtering using 0.22 µm filter. Cells were washed twice using 1× phosphate buffered saline (PBS) and fixed using 10% neutral buffered formalin for 30 min at room temperature. The cells were washed 3 times with 1× PBS followed by incubation with oil red O solution for 30 min at room temperature [55]. Cells were washed 5 times with distilled water and imaged by light microscopy. For quantitation of Oil Red O staining, cells were incubated with 100% isopropanol for 10 min, followed by measuring absorbance of the eluted stain at 500 nm.

### 4.5. Adenoviral Construct and Transduction

Adenoviruses containing hOGG1a with a mitochondrial targeting sequence (MTS) derived from manganese superoxide dismutase (*MnSOD*) and a GFP tag have been previously described [56] and were kindly provided by Dr. Lyudmila I. Rachek (University of South Alabama, USA). Adenoviruses were amplified in AD293 cells. Viral DNA was isolated and estimated using a Nanodrop to measure viral particle count (VPC). A VPC of 0.5 × 10^12^ VPC/cm^2^ resulted in a high and reproducible rate of transduction in 3T3-L1 CARΔ cells (Supplementary Figure S2A), without any apparent cytotoxicity, measured using the CellTiter-Blue^®^ Cell Viability Assay kit (Promega, Madison, WI, USA) (Supplementary Figure S2B). Accordingly, 0.5 × 10^12^ VPC/cm^2^ were added to the cells in growth medium in all further experiments. Cells were washed twice with 1× PBS and media was replaced with differentiation medium after 48 h of transduction. Transduction efficiency was assessed by analyzing the GFP positive cells using fluorescence microscopy for every experiment (Supplementary Figure S2A).

### 4.6. Western Blotting

At least three independent replicates of 3T3-L1 CARΔ cells were lysed in lysis buffer with protease and phosphatase inhibitors, followed by centrifugation at 600× *g* for 10 min and collection of supernatant as whole cell lysates. Protein concentration of pooled supernatants was determined using Bradford reagent. 30 µg of whole cell lysates were separated on 10% SDS-PAGE gels and transferred to PVDF membranes, followed by detection using appropriate primary and secondary antibodies. Cellular PARylation was determined by immunoblotting whole cell lysates using an anti-PAR antibody. Proteins were visualized by enhanced chemiluminescence or near-IR imaging. All protein blots were repeated at least twice, using independent replicates, to confirm results. Antibody sources are listed in Table 1.

### 4.7. Gene Expression

RNA for gene expression analysis was isolated using the Qiagen RNEasy kit. 1 µg of RNA was used to make cDNA using the Superscript III first-strand synthesis system (Thermo Fisher Scientific, Waltham, MA, USA). Gene expression was measured by quantitative real time PCR (qPCR) using gene specific primers on a Quant studio 3 Real-Time PCR system (Applied Biosystems, Foster City, CA, USA). Gene expression was normalized to expression of RNA18SN5. Primer sequences are listed in Table 2.

### 4.8. OGG1 Activity Assay

OGG1 activity was determined using whole cell lysates as previously described [57], with minor modifications. Briefly, a 40-mer oligonucleotide containing an 8-oxoG at position 19 and labeled at the 3′ end with indodicarbocyanine (5′AGAGAAGAAGAAGAA(G*)AGATGGGTTATTCGAACTAGCCy5Sp/3′) was hybridized to its complementary sequence containing a cytosine opposite the 8-oxoG lesion (G*) by heating at 65 °C for 15 min, followed by cooling at room temperature for 3 h. Total protein was isolated from cells without addition of protease and phosphatase inhibitors. 100 µg of whole cell lysate was incubated with 200 nM 8oxoG:C duplex in assay buffer containing 200 mM Tris-HCl pH 7.5, 20 mM EDTA, 1 mg/mL BSA, and 5% glycerol. The reaction mixture was incubated for 3 h at 37 °C, and the excision reaction was stopped by adding equal volume (50 µL) of gel loading buffer II (AM8547, Thermo Fisher) and heating at 95 °C for 5 min. 10 µL of the reaction was loaded on a 20% polyacrylamide gel containing 8M urea in Tris-borate-EDTA buffer, pH 8.4 to separate the excised DNA from its substrate. Separated bands were visualized using Azure c600 imager (Azure biosystems, USA).

### 4.9. Statistics

Data are expressed as mean  ±  SEM for biological replicates with two-group comparisons carried out using two-tailed student’s *t*-tests. *p*-values  <  0.05 were considered significant.

## Figures and Tables

**Table 1:** List of antibodies.

ANTIBODY NAME	SOURCE	CAT. NO.
OGG1	Abcam	62826
SCD1	Abcam	39969
PPAR©	Cell Signaling	2435S
β-ACTIN	Sigma-Aldrich	A5316
PARP-1	Pierce	MA5-15031
ANTI-PAR	Trevigen	4335-MC100
GAPDH	Cell Signaling	5174

**Table 2:** Primer sequences for gene expression studies.

GENE NAME	FORWARD PRIMER (5′ ⋙ 3′)	REVERSE PRIMER (5′ ⋙ 3′)
*mOgg1*	GCCAACAAAGAACTGGGAAA	CCCTCTGGCCTCTTAGATCC
*hOGG1*	GCTGGAGGCCGTGCGCAAGTAC	TGGGGTCTTGTCGCAGCAGTCG
*Scd1*	TGCGATACACTCTGGTGCTC	AGGATATTCTCCCGGGATTG
*Ppar-*©	AAGCCCATCGAGGACATCCA	CGGGTGGGACTTTCCTGCTA
*C/ebp-α*	CAAAGCCAAGAAGTCGGTGGACAA	TCATTGTGACTGGTCAACTCCAGC
*Pref-1*	GACCCACCCTGTGACCCC	CAGGCAGCTCGTGCACCCC
*Neil1*	GCCAGCCACTTTGTGAATGAG	AAGCTGAGATGTGGTAGGCAC
*Neil2*	GGGAGGCCCTCGTGGAT	TGTCCCGAAGCCAGTCCTT
*Neil3*	AAGTGATGGCAGCCCTCTGT	CCTCACAACTCGGAGAACACAA
*Nth1*	TGCTCTCCAGCCAGACCAA	CCCGGAGCCGTTGCA

## Data Availability

The data that support the findings of this study are available from the corresponding author upon reasonable request.

## Supplementary Materials

The following are available online at https://www.mdpi.com/1422-0067/22/3/1152/s1.
