# Supplementary material for: A Novel Role for the DNA Repair Enzyme 8-Oxoguanine DNA Glycosylase in Adipogenesis
Source: Int J Mol Sci. 2021 Jan 25;22(3):1152. doi: 10.3390/ijms22031152 (PMC7865743; doi:10.3390/ijms22031152)
Supplement: Supplementary file 1 [file ijms-22-01152-s001.pdf]

Supplementary InformatioN

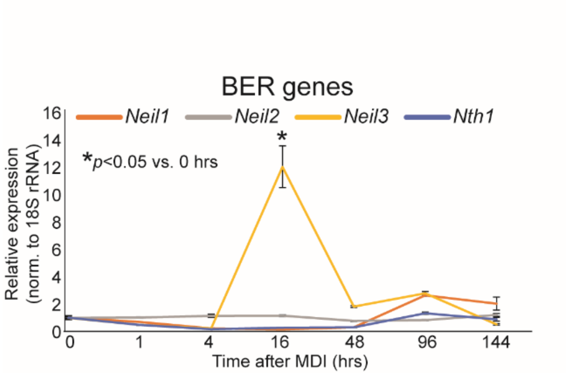

Fig. S1. Expression of BER genes during adipogenic differentiation. 3T3-L1 cells were differentiated using MDI. Cells were harvested and RNA and proteins were isolated at indicated time points. Gene expression of BER genes was measured by qRT-PCR using 18S rRNA as a control. Data are expressed as average ± SEM and represent at least 3 independent replicates per time point. *$p < 0.05$ vs 0 hr.

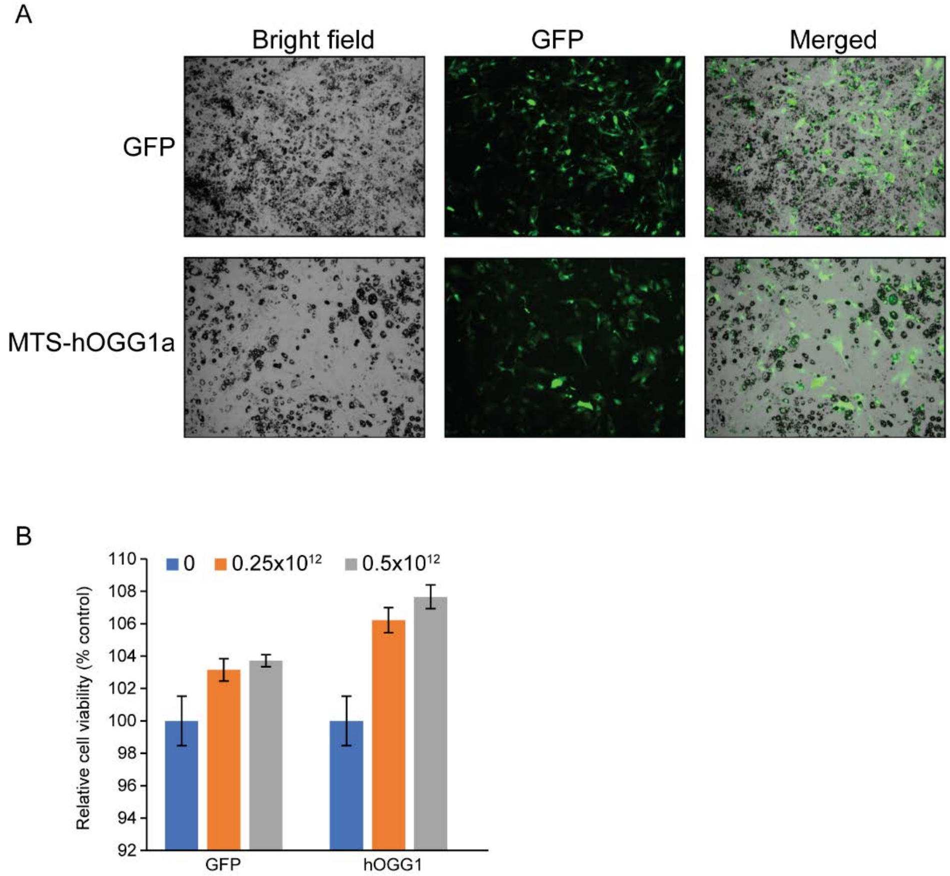

Fig. S2. Assessment of transduction efficiency and impact on cell viability. (A) 3T3-L1 CARΔ cells were transduced with 0.5×10^{12} VPC GFP or MTS-hOGG1a adenoviral particles, and transduction efficiency was assessed using phase contrast and fluorescence microscopy. (B) Cells were transduced with increasing concentrations of GFP or MTS-hOGG1a adenovirus particles and differentiated using MDI. Cell viability was assessed 8 days after transduction,

using the Cell Titer-Blue Cell Viability Assay. Data are expressed as average ± SEM and represent at least 3 independent replicates per time point.

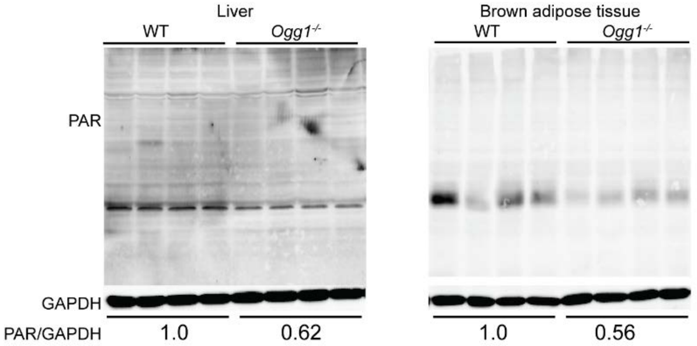

Fig. S3. Cellular PARylation in tissues from WT and *Ogg1*$^{-/-}$ mice. Cellular PARylation was assessed in protein extracts from liver and brown adipose tissue from WT vs. *Ogg1*$^{-/-}$ mice. Data are representative of 4 age- and sex-matched animals per genotype for tissue extracts. *$p < 0.05$ vs 0 hr.
